# Systemic immune-inflammation index predicts prognosis of patients with advanced pancreatic cancer

**DOI:** 10.1186/s12967-019-1782-x

**Published:** 2019-01-18

**Authors:** Ke Zhang, Yong-Qiang Hua, Dan Wang, Lian-Yu Chen, Cai-Jun Wu, Zhen Chen, Lu-Ming Liu, Hao Chen

**Affiliations:** 1 Department of Integrative Oncology, Fudan University Shanghai Cancer Center, Shanghai, 200032 China; 2 Department of Oncology, Shanghai Medical College, Fudan University, Shanghai, 200032 China

**Keywords:** Pancreatic cancer, Prognostic marker, Systemic immune-inflammation index, CA19-9

## Abstract

**Background:**

Systemic inflammation and immune dysfunction have been proved to be associated with cancer progression and metastasis in various malignancies. The aim of this retrospective study was to evaluate the prognostic significance of pre-treatment systemic immune-inflammation index (SII) in patients with advanced pancreatic cancer.

**Methods:**

In total, 419 patients diagnosed with advanced pancreatic cancer, between January 2011 and December 2015, were retrospectively enrolled. The SII was developed based on a training set of 197 patients from 2011 to 2013 and validated in an independent cohort of 222 patients from 2014 to 2015. Data on baseline clinicopathologic characteristics; pre-treatment laboratory variables such as absolute neutrophil, lymphocyte, and platelet counts; and carbohydrate antigen 19-9 (CA19-9), total bilirubin (TBIL), albumin (ALB), alkaline phosphatase (ALP), alanine transaminase (ALT), and aspartate transaminase (AST) levels were collected. The association between clinicopathologic characteristics and SII was assessed. The overall survival was calculated using the Kaplan–Meier survival curves and compared using the log-rank test. Univariate and multivariate Cox proportional hazard regression models were used to analyze the prognostic value of the SII.

**Result:**

An optimal cutoff point for the SII of 440 stratified the patients with advanced pancreatic cancer into high (> 440) and low (≤ 440) SII groups in the training cohort. Univariate and multivariate analyses revealed that the SII was an independent predictor for overall survival. The prognostic significance of the SII was confirmed in both normal and elevated CA19-9 levels.

**Conclusion:**

The baseline SII serves as an independent prognostic marker for patients with advanced pancreatic cancer and can be used in patients with both normal and elevated CA19-9 levels.

## Background

Pancreatic cancer is one of the most common malignant tumors and the seventh leading cause of cancer-related mortality worldwide [1]. In China, pancreatic cancer is the ninth type of cancer with the highest incidence and is ranked sixth in cancer-related mortality [2]. Surgical resection is the only curative treatment option; however, 80–85% of patients are diagnosed at advanced, inoperable stages [3]. Fluorouracil, leucovorin, irinotecan, and oxaliplatin (FOLFIRINOX) or gemcitabine-based chemotherapy is currently the standard treatment for these patients [4–6]. However, most patients do not respond or partially respond to the treatment, with a 5-year survival rate of < 5% [7]. Therefore, identifying the prognostic marker that helps to predict survival and guides the optimal therapy in patients with advanced pancreatic cancer is crucial.

Tumor-promoting inflammation has been recognized as an enabling characteristic of cancer [8]. The interplay between local immune response and systemic inflammation plays vital roles in cancer progression and patient survival [9]. The inflammatory response can be represented by the level of neutrophils, lymphocytes, platelets, and acute-phase proteins. These parameters are simple and easy to measure using standardized assays in clinical practice. Recently, neutrophils, lymphocytes, and platelets have been used in a joined tool, a systemic immune-inflammation index (SII), to obtain the prognostic information in patients with various malignant tumors, such as hepatocellular carcinoma, esophageal squamous cell carcinoma, gastric cancer, non-small-cell lung cancer, and colorectal cancer [10–14]. Mohammad et al. reported that SII is an independent predictor of cancer-specific survival and recurrence in resectable pancreatic cancer [15]. However, the significance of SII as a prognostic predictor in patients with advanced pancreatic cancer has not been examined.

Carbohydrate antigen 19-9 (CA19-9) is an extensively studied and validated serum biomarker with multiple clinical application in pancreatic cancer. It has a sensitivity and specificity of 79–81% and 82–90% for diagnosis in symptomatic patients [16, 17]; but is not useful as a screening marker because of low positive predictive value (0.5–0.9%) [18, 19]. CA19-9 also has prognostic and predictive value in resectable and advanced disease settings [20–23]. However, false negative results in Lewis negative genotype [24] and an increase false positive results in cases of biliary infection, inflammation, or biliary obstruction [25] may limit the prognostic role of serum CA19-9 in pancreatic cancer.

This study aimed to evaluate the prognostic value of SII in patients with advanced pancreatic cancer. Furthermore, based on different serum CA19-9 levels, the prognostic value of the SII was investigated separately.

## Patients and methods

### Ethics statement

This study was approved by the Ethics Committee of Fudan University Shanghai Cancer Center. All procedures were performed by the ethics standards of our institutional research committee and with those of the 1964 Helsinki Declaration and its later amendments or comparable ethical standards. Written informed consent was obtained from each participant by the institutional guidelines.

### Patient characteristics

A retrospective cohort study consisting of 419 patients diagnosed with advanced pancreatic cancer from January 2011 to December 2015 was conducted. All the patients were diagnosed and received primary treatment at Fudan University Shanghai Cancer Center. Patients who met the following criteria were enrolled in this study. (1) patients histologically or cytologically confirmed to have pancreatic adenocarcinoma and not recommended for curative resection; (2) those with stage III and IV tumors according to the 8th edition of the American Joint Committee on Cancer (Chicago, IL, USA) [26]; (3) those with no other primary malignant tumors found during treatment; and (4) those who did not have any hematologic disorder. The exclusion criteria included a lack of complete clinicopathologic and follow-up data, tumors not originating from the pancreas, and acute inflammatory diseases.

Our analyses involved two independent patient cohorts: the training cohort consisting of 197 patients diagnosed from January 2011 to December 2013 and the validation cohort consisting of 222 patients diagnosed from January 2014 to December 2015. The primary end-point of this study was overall survival (OS), which was defined as the interval between the diagnosis and death or the last follow-up. An independent researcher performed the follow-up work by conducting telephone interviews or reviewing medical records. Follow-up was terminated on December 31, 2016, in the training cohort and December 31, 2017, in the validation cohort.

### Clinical variables

Data on clinical variables, including demographic data, complete blood counts, tumor location, stage, CA 19-9 levels, and liver function parameters, such as total bilirubin (TBIL), albumin (ALB), alkaline phosphatase (ALP), alanine transaminase (ALT), and aspartate transaminase (AST) were collected. All laboratory parameters were assayed during routine workups before cancer diagnostic interventions. Data were extracted from the Electronic Medical Record System of Fudan University Shanghai Cancer Center.

### Serum assays for CA19-9

Baseline serum CA19-9 levels were examined within 1 week before the initiation diagnosis of pancreatic adenocarcinoma. Serum CA19-9 levels were detected using an electrochemiluminescence immunoassay on the Roche Cobas e601 immunoassay analyzer (Roche Diagnostics, Mannheim, Germany). Normal CA19-9 was defined as CA19-9 level lower than 37 U/mL [27].

### Systemic immune-inflammation index

The SII is an index based on the platelet (P), neutrophil (N), and lymphocyte (L) counts and calculated using the following formula: SII = P*N/L as defined previously [10].

The X-tile 3.6.1 software (Yale University, New Haven, CT) was used for bioinformatic analysis to determine the optimal cutoff value of SII in the training cohort [28]. Consequently, the SII scores were stratified into ≤ 440*10^9^ or > 440*10^9^ for all subsequent analyses.

### Statistical analysis

Continuous variables were presented as the median and range. Cumulative survival rates were calculated using the Kaplan–Meier method, and between-group differences were assessed using the log-rank test. Univariate and multivariate analyses were calculated using the Cox proportional hazards regression model. Pearson’s Chi square test was used to compare groups. A *P* value of < 0.05 was considered to be statistically significant.

## Results

### Patient characteristics

The clinical characteristics of patients in the training and validation cohorts are shown in Table 1. For the whole study population, 269 (64.2%) were men and 150 (35.8%) women, with the median age of 61 (range 25–84) years. Additionally, 88 patients (27.5%) were diagnosed with locally advanced disease, and the remaining 232 (72.5%) were diagnosed with metastatic disease. In the training cohort, all patients died during the last follow-up, with a median OS of 6.6 months. In the validation cohort, six out of 219 patients survived during the last follow-up, with a median OS of 8.7 months. The SII of > 440 was observed in 53.8% (106/197) of the patients in the training cohort and 59.9% (133/222) in the validation cohort. As shown in Table 1, the clinicopathologic characteristics were similar between the two cohorts.

**Table 1:** The clinicopathologic characteristics of patients in the training and validation cohorts

Variables	Training		Validation		P
	N = 197	%	N = 222	%	
Age (years)					
≤ 60	95	48.2	105	47.3	0.922
> 60	102	51.8	117	52.7	
Sex					
Male	122	61.9	147	66.2	0.414
Female	75	38.1	75	33.8	
Location					
Head	90	40.5	87	44.2	0.488
Body/tail	132	59.5	110	55.8	
Metastasis					
No	41	20.8	42	18.9	0.713
Yes	156	79.2	180	81.1	
CA19-9 (U/mL)					
≤ 37	44	22.3	52	23.4	0.817
> 37	153	77.7	170	76.6	
TBIL (μmol/L)					
≤ 17	162	82.2	184	82.9	0.898
> 17	35	17.8	38	17.1	
ALB (g/L)					
≤ 35	13	6.6	13	5.9	0.840
> 35	184	93.4	209	94.1	
ALP (U/L)					
≤ 125	139	70.6	157	70.7	1.000
> 125	58	29.4	65	29.3	
ALT (U/L)					
≤ 35	148	75.1	172	77.5	0.645
> 35	49	24.9	50	22.5	
AST (U/L)					
≤ 40	165	83.8	186	83.8	1.000
> 40	32	16.2	36	16.2	

*CA* carbohydrate antigen, *TBIL* total bilirubin, *ALB* albumin, *ALP* alkaline phosphatase, *ALT* alanine transaminase, *AST* aspartate transaminase

### Association between the SII and clinicopathologic parameters

The correlation between the SII and clinicopathologic parameters is shown in Table 2. In the training cohort, patients with an SII of > 440 were more likely to have metastasis (P = 0.014) and low ALB levels (P = 0.023). In the validation cohort, an SII of > 440 was associated with high ALP levels (P = 0.016).

**Table 2:** The correlation between SII and clinicopathologic characteristics in training and validation cohorts

Variables	Training (N = 197)					Validation (N = 222)				
	SII ≤ 440	%	SII > 440	%	P	SII ≤ 440	%	SII > 440	%	P
Age (years)										
≤ 60	39	42.9	56	52.8	0.198	41	46.1	64	48.1	0.785
> 60	52	57.1	50	47.2		48	53.9	69	51.9	
Sex										
Male	55	60.4	67	63.2	0.769	62	69.7	85	63.9	0.389
Female	36	39.6	39	36.8		27	30.3	48	36.1	
Location										
Head	34	37.4	53	50	0.085	38	42.7	52	39.1	0.676
Body/tail	57	62.6	53	50		51	57.3	81	60.9	
Metastasis										
No	26	28.6	15	14.2	0.014	19	21.3	23	17.3	0.487
Yes	65	71.4	91	85.8		70	78.7	110	82.7	
CA19-9 (U/mL)										
≤ 37	22	50.0	22	50.0	0.609	22	42.3	30	57.7	0.748
> 37	69	45.1	84	54.9		67	39.4	103	60.6	
TBIL (μmol/L)										
≤ 17	80	87.9	82	77.4	0.062	77	86.5	107	80.5	0.278
> 17	11	12.1	24	22.6		12	13.5	26	19.5	
ALB (g/L)										
≤ 35	2	2.2	11	10.4	0.023	5	5.6	8	6.0	1.000
> 35	89	97.8	95	89.6		84	94.4	125	94.0	
ALP (U/L)										
≤ 125	70	76.9	69	65.1	0.085	71	79.8	86	64.7	0.016
> 125	21	23.1	37	34.9		18	20.2	47	35.3	
ALT (U/L)										
≤ 35	69	75.8	79	74.5	0.870	72	80.9	100	75.2	0.332
> 35	22	24.2	27	25.5		17	19.1	33	24.8	
AST (U/L)										
≤ 40	76	83.5	89	84.0	1.000	80	89.9	106	79.7	0.062
> 40	15	16.5	17	16.0		9	10.1	27	20.3	

*CA* carbohydrate antigen, *TBIL* total bilirubin, *ALB* albumin, *ALP* alkaline phosphatase, *ALT* alanine transaminase, *AST* aspartate transaminase

### Prognostic significance of the SII in the training cohort

Univariate analysis indicated that metastasis, CA19-9, TBIL, and SII were prognostic factors for OS in the training cohort, whereas age, gender, tumor location, ALB, ALP, ALT, and AST had no prognostic value for OS (Table 3). A high SII was significantly associated with shorter OS (hazard ratio [HR] = 1.549, 95% confidence interval [CI] = 1.16–2.06, P = 0.003).

**Table 3:** Univariate Cox regression analyses of the SII with clinicopathologic characteristics [training cohort (n = 197) and validation cohort (n = 222)]

Variables	Training		Validation	
	HR (95% CI)	P	HR (95% CI)	P
Age, years (≤ 60 vs. > 60)	1.238 (0.93–1.64)	0.139	0.907 (0.69–1.19)	0.474
Sex (male vs. female)	1.234 (0.92–1.65)	0.154	1.011 (0.76–1.34)	0.942
Location (head vs. body/tail)	0.845 (0.64–1.12)	0.243	0.933 (0.71–1.23)	0.620
Metastasis (no vs. yes)	1.580 (1.11–2.26)	0.012	1.686 (1.18–2.41)	0.004
CA19-9, U/mL (≤ 37 vs. > 37)	1.580 (1.12–2.23)	0.009	1.191 (0.87–1.64)	0.285
TBIL, μmol/L (≤ 17 vs. > 17)	1.578 (1.09–2.28)	0.015	1.472 (1.03–2.10)	0.033
ALB, g/L (≤ 35 vs. > 35)	0.722 (0.41–1.27)	0.259	0.644 (0.36–1.16)	0.140
ALP, U/L (≤ 125 vs. > 125)	1.101 (0.81–1.50)	0.540	1.272 (0.94–1.72)	0.118
ALT, U/L (≤ 35 vs. > 35)	1.061 (0.77–1.47)	0.719	1.090 (0.79–1.50)	0.598
AST, U/L (≤ 40 vs. > 40)	1.019 (0.70–1.49)	0.922	1.437 (1.00–2.07)	0.052
SII (≤ 440 vs. > 440)	1.549 (1.16–2.06)	0.003	1.458 (1.11–1.92)	0.008

*CA* carbohydrate antigen, *TBIL* total bilirubin, *ALB* albumin, *ALP* alkaline phosphatase, *ALT* alanine transaminase, *AST* aspartate transaminase, *SII* systemic immune-inflammation index

**Fig. 1:**
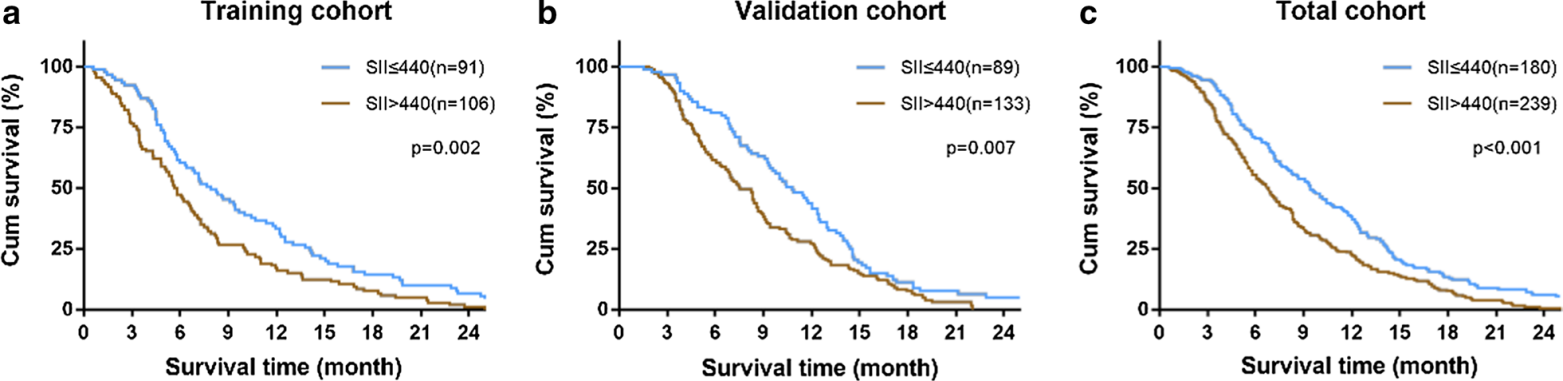
Prognostic significance of SII in patients with advanced pancreatic cancer. Kaplan–Meier analysis of OS for SII in the training (**a**), validation (**b**), and total cohorts (**c**)

The Kaplan–Meier analysis indicated that higher SII was associated with shorter OS (P = 0.002, Fig. 1a). The median OS was 7.9 months and 5.7 months for patients with SII of ≤ 440 and SII of > 440, respectively. Based on the result of our multivariate analysis, the SII was an independent prognostic factor for OS (HR = 1.502, 95% CI = 1.13-2.00, P = 0.005, Table 4).

**Table 4:** Multivariate Cox regression analyses of the SII in the training and validation cohorts

Variables	Training		Validation	
	HR (95% CI)	P	HR (95% CI)	P
Metastasis (no vs. yes)	1.318 (0.91–1.91)	0.144	1.696 (1.19–2.42)	0.004
CA19-9, U/mL (≤ 37 vs. > 37)	1.521 (1.08–2.15)	0.017	NA	NA
TBIL, μmol/L (≤ 17 vs. > 17)	1.325 (0.91–1.93)	0.145	1.509 (1.06–2.16)	0.024
SII (≤ 440 vs. > 440)	1.502 (1.13–2.00)	0.005	1.455 (1.10–1.92)	0.008

*CA* carbohydrate antigen, *TBIL* total bilirubin, *SII* systemic immune-inflammation index

### Validation of the SII in an independent cohort

The prognostic value of the SII was confirmed in an independent validation cohort of 222 patients. The results were similar with those obtained from the training cohort (Table 3). The Kaplan–Meier analysis showed that the high SII was associated with shorter OS in the validation cohort (P = 0.007, Fig. 1b) and total cohort (P < 0.001, Fig. 1c). Univariate and multivariate analyses demonstrated that the SII was significantly correlated with OS (HR = 1.455, 95% CI = 1.10–1.92, P = 0.008, Table 4).

### Prognostic significance of the SII in patients with normal and elevated CA19-9 levels

**Fig. 2:**
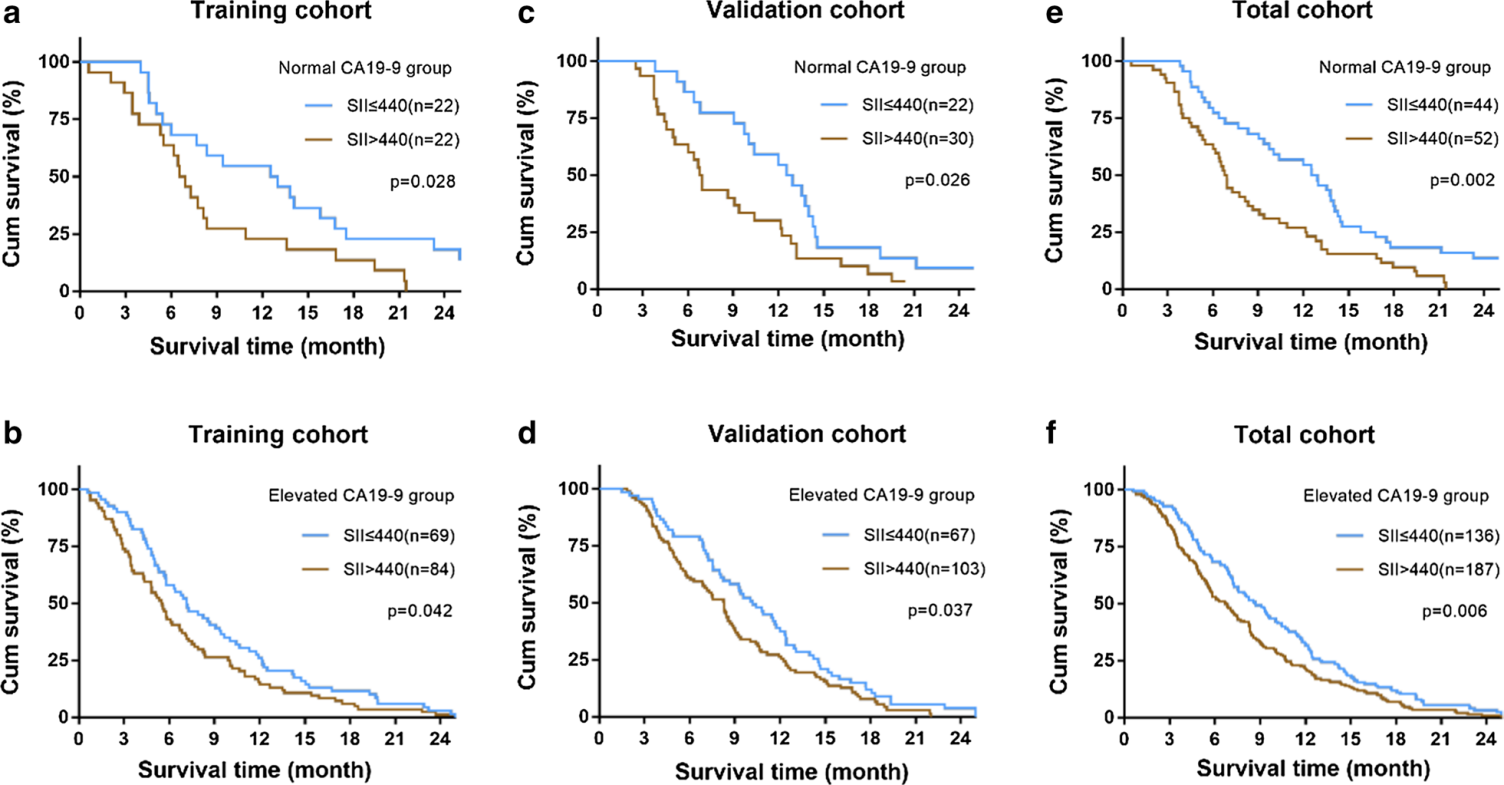
Prognostic significance of SII in patients with normal and elevated CA19-9. Kaplan–Meier analysis of OS for SII in normal and elevated CA19-9 groups in the training (**a**, **b**), validation (**c**, **d**), and total cohorts (**e**, **f**)

We further investigated the prognostic significance of the SII according to different CA19-9 levels. We found that the SII score was significantly correlated with OS in both normal and elevated CA19-9 groups in the training cohort (P = 0.028, Fig. 2a and P = 0.042, Fig. 2b). The prognostic value for OS in normal and elevated CA19-9 groups was also confirmed in the validation cohort (P = 0.026, Fig. 2c and P = 0.037, Fig. 2d) and total cohorts (P = 0.002, Fig. 2e and P = 0.006, Fig. 2f).

## Discussion

Systemic inflammation is an essential promoter of proliferation, invasion, and metastasis of tumor cells [29, 30]. The immune system also plays a vital role in cancer surveillance and elimination [31]. In this study, we constructed an immune-inflammation-based prognostic score (SII) based on the peripheral lymphocyte, neutrophil, and platelet counts. Furthermore, we demonstrated SII as a predictor of survival in patients with advanced pancreatic cancer in two independent cohorts. Also, the SII maintained its prognostic significance in both normal and elevated CA19-9 levels.

The relationship between SII and advanced pancreatic cancer prognosis may be due to the high SII results from thrombocythemia, neutrophilia, and lymphopenia, which suggests an elevated inflammatory status and decreased immune system response. Cancer inflammation has been known to have a normal impact on survival [31, 32]. Increasing evidence has shown that neutrophilia and thrombocythemia are associated with pro-cancer effects [33–36]. Neutrophils can not only enhance cancer cell invasion, proliferation, and metastasis but also assist the evasion of cancer cells on immune surveillance [37]. Platelets can guard tumor cells against immune elimination and promote their arrest at the endothelium, supporting the establishment of secondary lesions [38]. For example, in pancreatic cancer, platelets support the adhesion of tumor cells to escape from the host’s immune surveillance. Circulating tumor cells (CTCs) are neoplastic cells shed from a solid tumor into the bloodstream and associated with tumor metastases [39] [40]. Platelets can also protect the CTCs from shear stresses during circulation, inducing CTC epithelial-mesenchymal transition [41]. Our results showed that patients with high SII in the training cohort were more likely to have metastatic disease.

Conversely, lymphocytes are known to play a crucial role in tumor defense by inducing cell death and inhibiting cell proliferation and migration [42]. Lymphopenia, which indicates the ineffectiveness of the immune surveillance systems, is also observed in pancreatic cancer [43] and has been reportedly associated with poor survival in several malignant tumors [44]. Considering these reasons, patients with higher SII presented with poor survival. These results help us to better understand the role of neutrophils, platelets, and lymphocytes in cancer and their relationship with immunity and inflammation. Also, patients with advanced pancreatic cancer who have a high SII might benefit from targeted anti-inflammatory agents, such as aspirin [45]. Recently, immune checkpoint inhibitors have been approved for the treatment of various cancer, which urges the development of immune-specific biomarkers. Previous studies have reported the prognostic value of SII in patients undergoing immunotherapy [46]. Therefore, the SII may serve as a prognostic marker in immunotherapy.

CA19-9 is a sialylated Lewis A blood group antigen and commonly expressed and shed in pancreatic and hepatobiliary disease and in many malignancies. Although CA19-9 is a well-established serum biomarker to predict the prognosis of pancreatic cancer [47, 48], it is important to note that CA19-9 may be undetectable in Lewis antigen-negative individuals [24]. In our study, approximately a quarter of the patients with advanced pancreatic cancer presented with normal CA19-9 levels (≤ 37 U/mL) upon the diagnosis. Furthermore, CA19-9 may be falsely positive in cases of biliary infection or biliary obstruction [25]. In our study, about 17% patients presented with high total bilirubin (> 17 μmol/L). In addition, the level of CA19-9 was also influenced by age and gender [49]. In consequence, the prognostic value of CA19-9 in pancreatic cancer is limited. Our results showed that SII serves as an independent prognostic factor in not only normal CA19-9 group but also elevated CA19-9 group. This suggests that SII complements with CA19-9 in predicting the outcomes in patients with advanced pancreatic cancer.

This study has a few limitations. First, this was a retrospective study performed in a single center; thus, multicenter studies should be performed to provide stronger evidence. Second, further studies should be conducted to explore the underlying mechanism between SII and cancer biology. Third, although the prognostic value of the SII was confirmed, we did not compare the discrimination ability of the SII with other prognostic markers.

## Conclusion

In conclusion, 419 patients with advanced pancreatic cancer were retrospectively enrolled, and the prognostic significance of the SII was determined in two independent cohorts. Our result confirmed that the SII could serve as an independent prognostic marker in patients with advanced pancreatic cancer. Also, the SII can be used in patients with both normal and elevated CA19-9 levels.

## Abbreviations

SII: systemic immune-inflammation index
CA19-9: carbohydrate antigen 19-9
TBIL: total bilirubin
ALB: albumin
ALP: alkaline phosphatase
ALT: alanine transaminase
AST: aspartate transaminase
OS: overall survival
HR: hazard ratio
CI: confidence interval
